# The Microbial Community in the Feces of the White Rhinoceros (*Ceratotherium simum*) as Determined by Barcoded Pyrosequencing Analysis

**DOI:** 10.1371/journal.pone.0070103

**Published:** 2013-07-26

**Authors:** Gaorui Bian, Li Ma, Yong Su, Weiyun Zhu

**Affiliations:** Laboratory of Gastrointestinal Microbiology, College of Animal Science and Technology, Nanjing Agricultural University, Nanjing, P. R. China; Wageningen University, The Netherlands

## Abstract

As a non-ruminant herbivore, the white rhinoceros has the ability to utilize fibrous plant matter through microbial fermentation in the hindgut. So far, there has been no report using molecular techniques to study the gut microbiota of the white rhinoceros. We used barcoded pyrosequencing to characterize 105,651 sequences of 16S rRNA genes obtained from fecal samples from five white rhinoceroses. Results showed that Firmicutes and Bacteroidetes were the predominant phyla in the samples, which were comprised largely of unclassified bacteria. The microbiota of one animal treated with drug therapy differed from those in other healthy animals, and was dominated by *Aerococcus -*related bacteria. The core microbiota in the healthy rhinoceros were dominated by phyla Firmicutes and Bacteroidetes, represented by the *Ruminococcaceae*, *Lachnospiraceae*, *Rikenellaceae* and *Prevotellaceae* families. The present work provides a phylogenetic framework for understanding the complex microbial community of the rhinoceros; however, further studies are required to link the distinctive microbiota with their digestive role in the hindgut of the white rhinoceros.

## Introduction

The rhinoceros is one of five surviving species of odd-toed ungulates in the *Rhinocerotidae* family. The five different species of rhinoceros include two African species, the white rhinoceros (*Ceratotherium simum*) and the black rhinoceros (*Diceros bicornis*), and three Asian species, Indian rhinoceros (*Rhinoceros unicornis)*, Sumatran rhinoceros (*Dicerorhinus sumatrensis*), and Javan rhinoceros (*Rhinoceros sondaicus*). Relative to the four other species which are in the list of endangered wild animals, the white rhinoceros is classed as vulnerable, with roughly 16,000 remaining in the wild in 2007 (IUCN 2008).

The white rhinoceros is, after the elephant, the largest extant mammalian herbivore [1]. As a hindgut fermenter, the white rhinoceros has the ability to utilize fibrous plant matter through microbial fermentation in the hindgut. Comparative studies among non-ruminant herbivores showed that the rhinoceros had a similar digestive system to horses and elephants [2], [3]. Costa et al. found that Firmicutes predominated (68%) in the feces of healthy horses, followed by Bacteroidetes (14%) and Proteobacteria (10%) [4]. At the genus level, previous studies showed that cellulose-digesting microflora (e.g., *Ruminococcus* and *Fibrobacter* species) were important members of the microbial community in the rumen or the hindgut of non-ruminant herbivores [5], [6], which enabled the host to gain nutrients from fibrous plant materials. However, information on microbial diversity in the hindgut of the white rhinoceros remains limited. To our knowledge, there has been no report using molecular techniques to study microbial flora in the feces of white rhinoceros.

As a specialized grazing species (focusing on leaves and grass), the white rhinoceros is able to eat plants that are toxic to other animals. To understand whether this animal has distinctive gut microbiota, and whether the tolerance of the white rhinoceros to toxicants is related to gut microbiota, comprehensive analysis of the bacterial community is required. The development of high throughput sequencing has led to a revolution in the characterization of complex microbial populations [4], [7], [8]. Thus, the aim of this study was to investigate the microbial community in the feces of white rhinoceroses using the high throughput pyrosequencing analysis.

## Results

**Figure 1:**
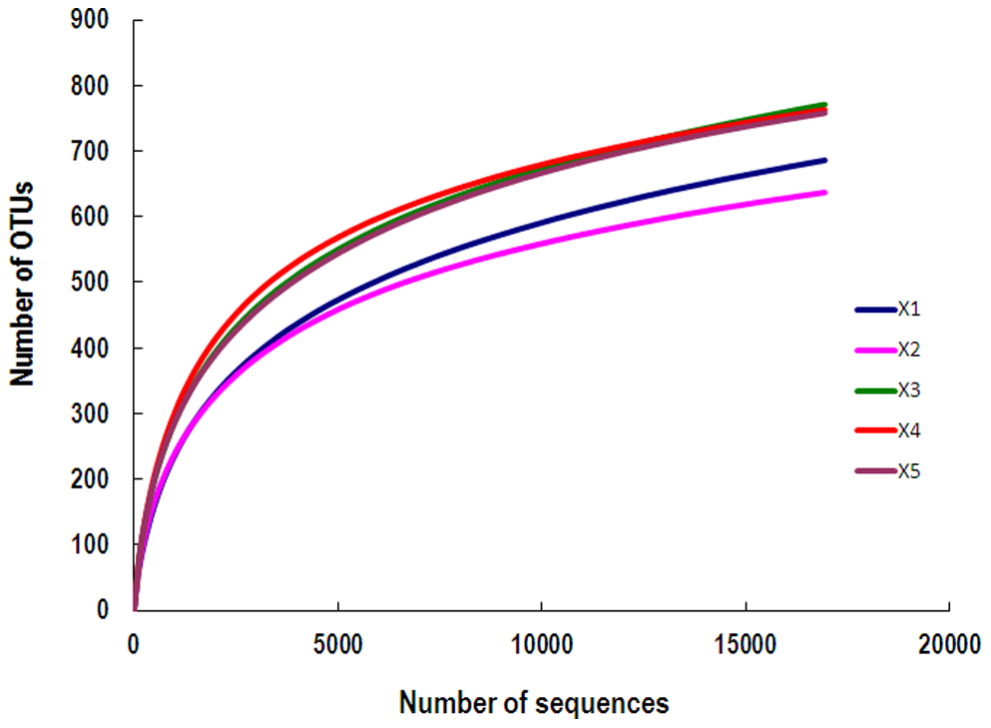
Rarefaction curves. Rarefaction curves comparing the number of reads with the number of phylotypes found in the DNA in the feces of five rhinoceroses.

Across all five samples, 105,651 quality sequences from 116,208 reads were classified as bacteria. The average length of quality sequences was 482 bp. The total number of sequences, coverage, the number of OTUs, and statistical estimates of species richness for 16,929-sequence subsets from each sample at a genetic distance of 3% are presented in Table 1. The rarefaction curves generated by MOTHUR plotting the number of reads by the number of OTUs tended to approach the saturation plateau (Figure 1). Libshuff analysis indicated that differences in the bacterial community structure between the library of X1 and libraries of other animals were significant (*P<*0.001).

**Table 1:** Phylotype coverage and diversity estimation of the 16S rRNA gene libraries of the feces of rhinoceroses from the pyrosequencing analysis^1^.

Rhinoceros	Reads	OTUs	ACE	Chao	Shannon	Simpson	coverage
X1	16,929	686	861.4	903.4	4.649	0.0355	0.9890
X2	16,929	636	760.5	786.0	4.762	0.0284	0.9912
X3	16,929	765	946.4	1033.7	5.254	0.0130	0.9885
X4	16,929	762	890.5	946.7	5.300	0.0131	0.9904
X5	16,929	757	901.9	917.6	5.282	0.0122	0.9900

1 The operational taxonomic units (OTUs) were defined with 3% dissimilarity. The coverage percentages, richness estimators (ACE and Chao), and diversity indices (Shannon and Simpson) were calculated.

### Taxonomic Composition

**Figure 2:**
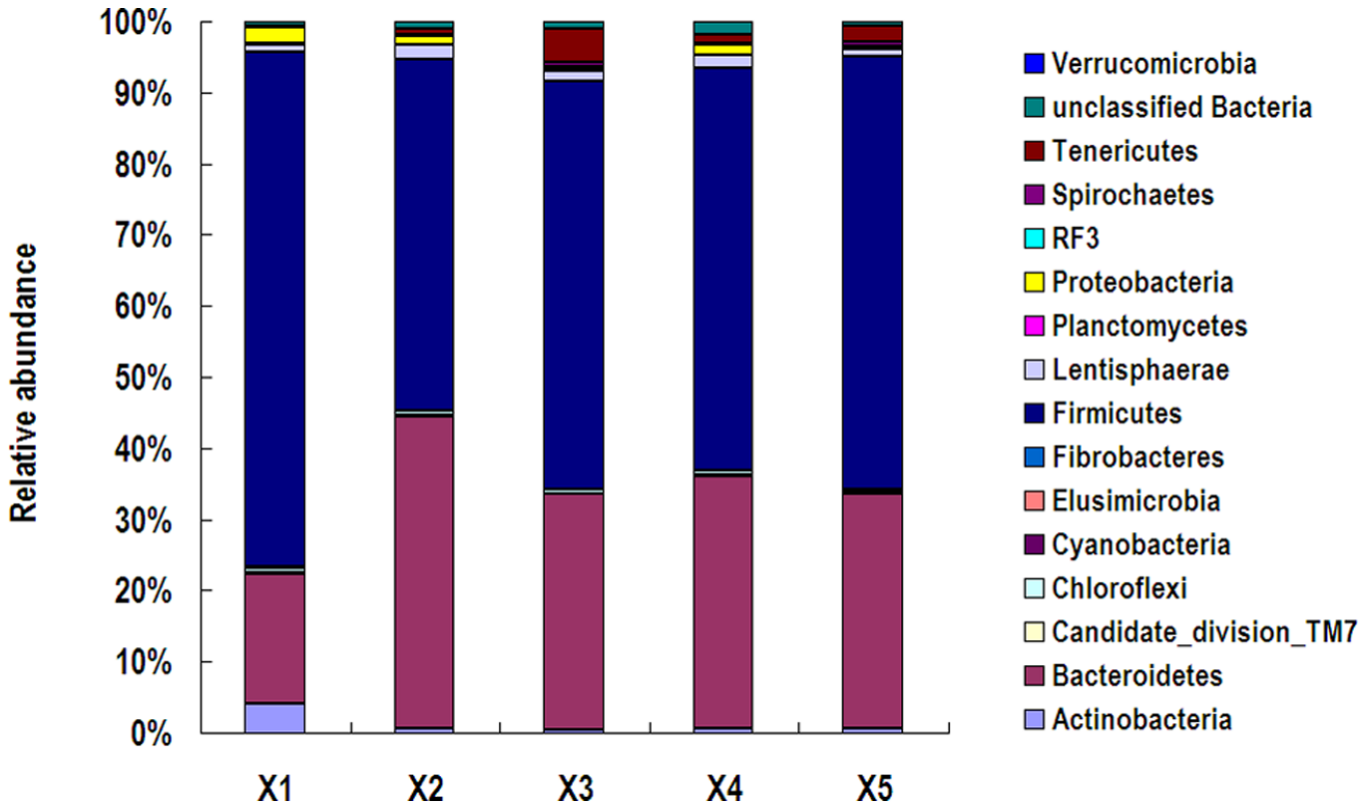
Fecal bacterial community at the phylum level. Relative abundance of bacterial groups (phylum level) in the feces of five white rhinoceroses.

A total of 16 prokaryotic phyla were identified from the 16S rRNA gene sequences (Figure 2). In the feces of five rhinoceroses, Firmicutes were predominant, represented by 49.48%-72.52% of 16S rRNA gene sequences. Bacteroidetes was the second most abundant phylum at 18.18%-43.83%. These two phyla were more than 90% of the total sequences in all five animals. A high relative abundance of Actinobacteria (4.10%) was found in rhinoceros X1 compared to the other four animals (lower than 0.7%). In contrast, rhinoceros X1 had a lower abundance of Bacteroidetes (18.18%) in its feces than the other four animals (33.05%-43.83%).

**Figure 3:**
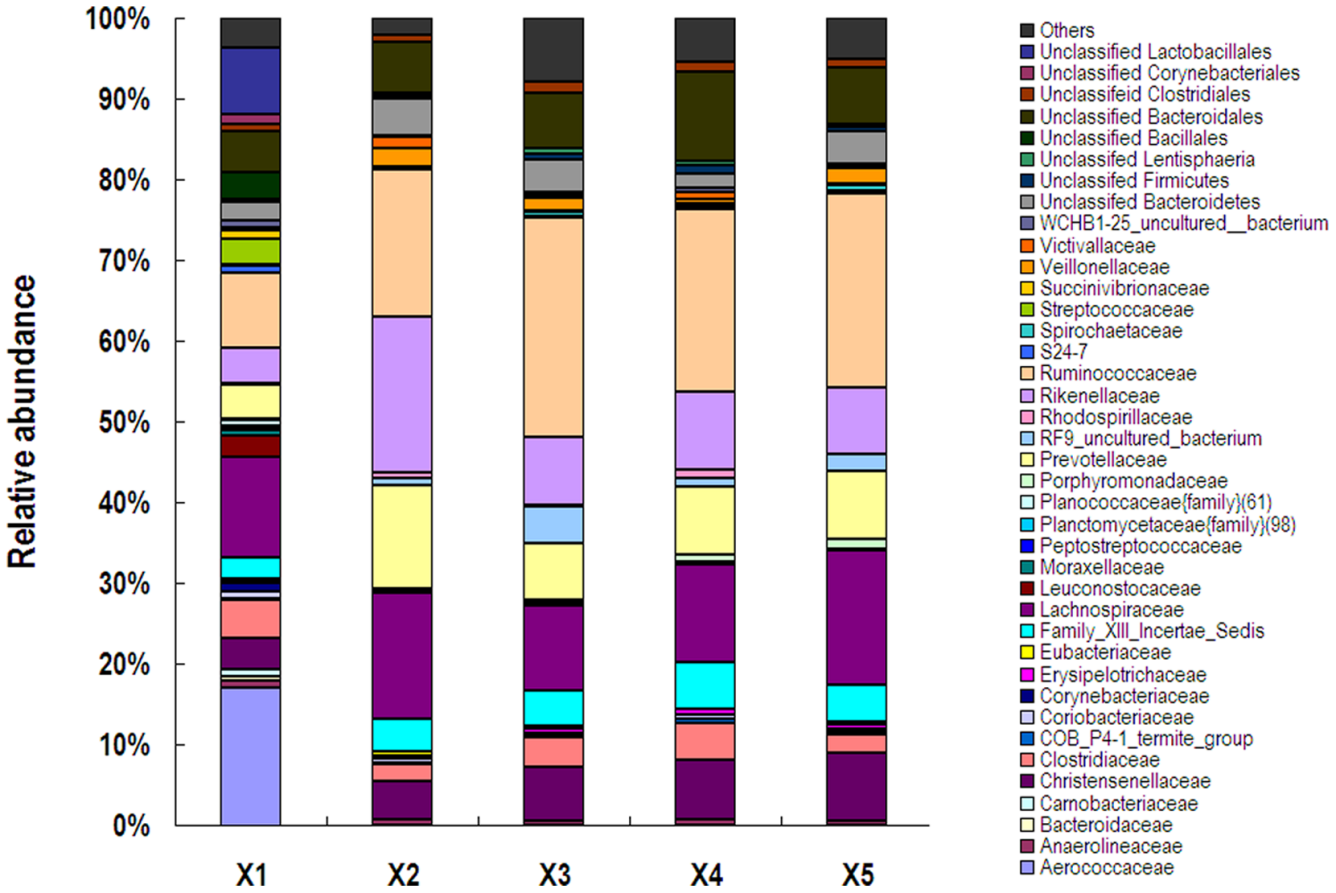
Fecal bacterial community at the family level. Relative abundance of bacterial groups (family level) in the feces of five white rhimoceroses.

At the family level, the abundances of unclassified bacteria in the samples from X1, X2, X3, X4 and X5 were 42.23%, 14.56%, 21.66%, 21.01% and 18.10%, respectively (Figure 3). In the feces of X1, *Aerococcaceae* was predominant with the abundance of 17.10%, followed by *Lachnospiraceae* and *Ruminococcaceae* and unclassified *Lactobacillales*. In the feces of other four healthy animals, the most abundant families were *Ruminococcaceae*, *Lachnospiraceae*, *Rikenellaceae*, *Prevotellaceae* and unclassified *Bacteroidales*, which made up approximantely 65% (59.70% to 72.22%) of total sequences.

**Figure 4:**
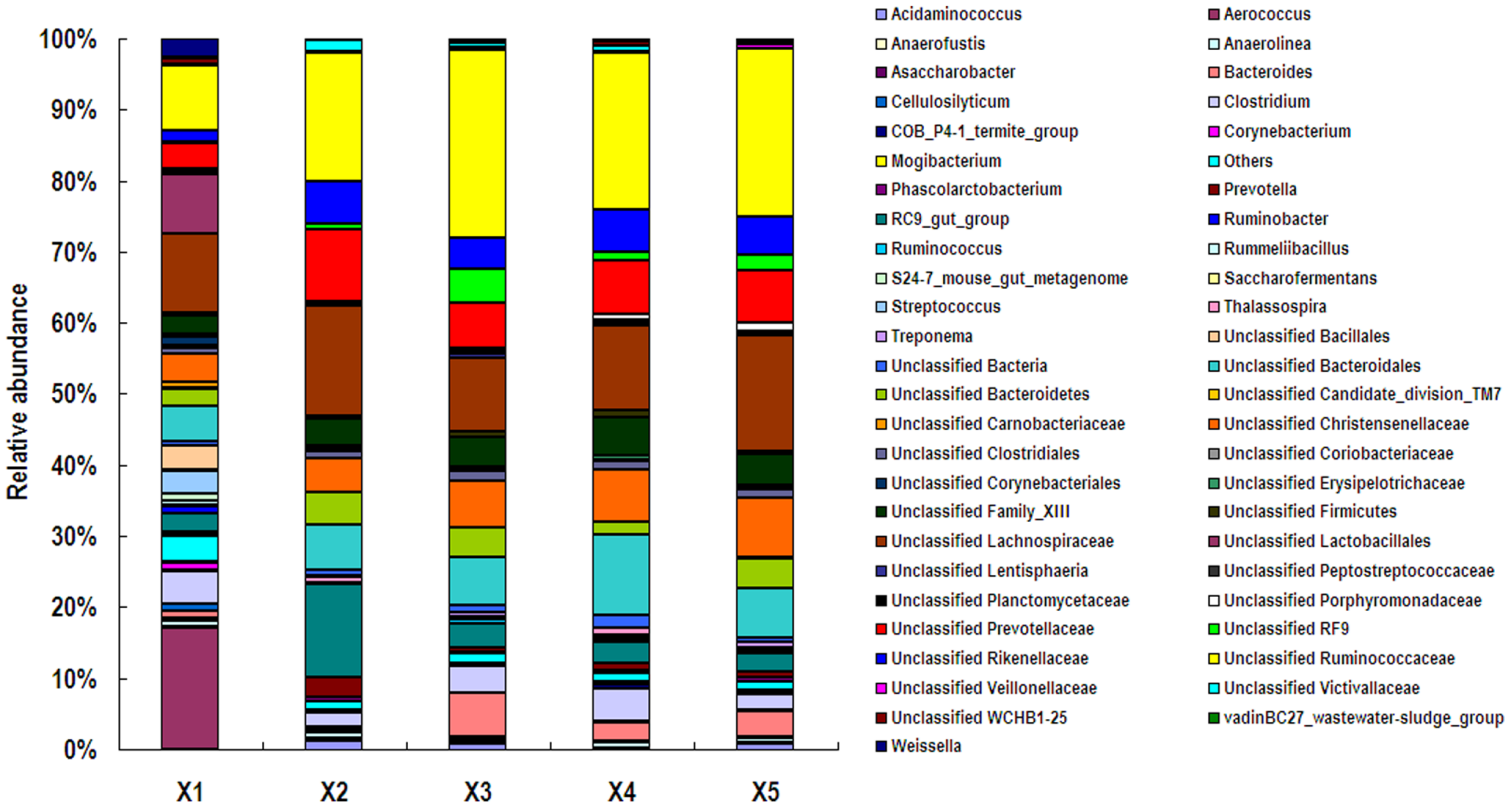
Fecal bacterial community at the genus level. Relative abundance of bacterial groups (genus level) in the feces of five white rhinoceroses.

**Figure 5:**
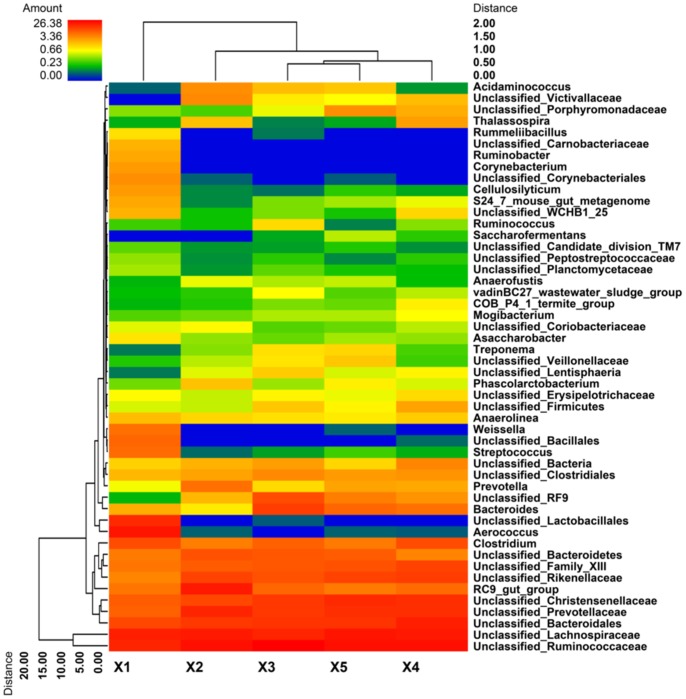
Bacterial distribution among the five samples. Double dendrogram showing the bacterial distribution among the fecal samples of five rhinoceroses. The bacterial phylogenetic tree was calculated using the neighbor-joining method, and the relationship among samples was determined using Bray distance and the complete clustering method. Total 50 genera with the abundance higher than 0.1% within total bacteria were sorted for the analysis. The heatmap plot depicts the relative percentage of each bacterial genus (variables clustering on the Y-axis) within each sample (X-axis clustering). The relative values for the bacterial genus are depicted by color intensity in the legend indicated at the top of the figure. Clusters based on the distance of the five samples along the X-axis and the bacterial genera along the Y-axis are indicated at the top and bottom of the figure, respectively.

At the genus level, 57.94% of total reads classified as bacteria in the feces of rhinoceros X1 were unclassified, while the abundances of unclassified bacteria in the samples from X2, X3, X4, and X5 were higher, approximantely 80% (75.40% to 84.78%) (Figure 4). In the feces of X1, genus *Aerococcus* was predominant with the abundance higher than 17%, followed by unclassified *Lachnospiraceae*, unclassified *Ruminococcaceae*, and unclassified *Lactobacillales*. In the feces of X2, the most abundant genera were unclassified *Ruminococcaceae*, unclassified *Lachnospiraceae*, RC9 gut group, unclassified *Prevotellaceae*, unclassified *Bacteroidales*, and unclassified *Rikenellaceae*, which made up 68.9% of total sequences. Unclassified *Ruminococcaceae*, unclassified *Lachnospiraceae*, and unclassified *Bacteroidales* were the three most predominant groups in the feces of X3, X4, and X5. Genera *Corynebacterium* and *Ruminobacter* were only observed in samples of X1 with abundances of 1.04% and 0.93% of total bacteria, respectively. The abundances of genera *Aerococcus*, *Corynebacterium*, and *Weissella* in the feces of X1 were more than 200 times higher than those of other animals. Clustered heatmap analysis based on the bacterial community profiles at the genus level disclosed that samples from animals X3, X4 and X5 were grouped together with a similarity higher than 70%, while X1 was outlier from the other four animals (Figure 5).

As shown in Table 2, *Aerococcus- viridans* related OTU dominated in the X1 library with the abundance of 15.24%. *Lactobacillales* and *Bacillales-*related OTUs, which were only found in the X1 sample, represented 8.08% and 3.30% of sequences in the X1 library. *Prevotellaceae*, *Ruminococcaceae*, *Lachnospiraceae*, *Bacteroidales* and *Clostridium*-related OTUs predominated in all five libraries.

**Table 2:** Relative abundance of predominant OTUs (percentage) in the feces of five African white rhinoceroses^1^.

OTUs	Rhinoceros					Annotation^2^
	X1	X2	X3	X4	X5	
OTU1	1.365	7.591	4.495	4.141	5.718	f: *Prevotellaceae*
OTU3	1.819	11.944	1.932	0.898	0.904	g: RC9_gut_group
OTU4	3.763	1.979	3.544	4.519	2.168	g: *Clostridium*
OTU2	15.240	0.012	0.000	0.000	0.012	s: *Aerococcus viridans*
OTU8	0.662	0.189	5.464	2.416	2.936	g: *Bacteroides*
OTU6	1.500	0.703	2.197	5.216	1.069	o: *Bacteroidales*
OTU12	2.339	2.688	1.802	0.768	2.268	o: *Bacteroidales*
OTU9	0.000	3.704	1.170	0.012	3.905	f: *Lachnospiraceae*
OTU13	0.721	0.106	1.223	2.889	2.776	f: *Christensenellaceae*
OTU5	0.112	1.465	2.127	0.490	2.570	f: *Ruminococcaceae*
OTU10	0.219	1.181	1.022	2.505	1.506	f: *Rikenellaceae*
OTU23	0.000	0.295	1.459	2.989	1.394	o: *Bacteroidales*
OTU19	0.006	1.802	1.559	2.132	0.419	f: *Ruminococcaceae*
OTU11	0.000	0.461	2.865	0.000	2.056	p: Bacteroidetes
OTU14	5.381	0.000	0.000	0.000	0.000	o: *Lactobacillales*
OTU15	0.254	3.237	0.916	0.059	0.862	f: *Lachnospiraceae*
OTU49	2.262	0.502	0.089	1.175	1.235	p: Bacteroidetes
OTU18	0.106	0.892	2.121	0.603	1.081	f: *Ruminococcaceae*
OTU55	0.425	0.656	1.412	0.939	1.270	f: *Ruminococcaceae*
OTU7	0.366	0.431	0.951	1.967	0.981	f: *Prevotellaceae*
OTU20	0.000	3.343	0.821	0.000	0.484	p: Bacteroidetes
OTU16	0.354	0.679	0.715	1.388	1.370	f: *Ruminococcaceae*
OTU25	1.760	0.242	0.443	1.240	0.443	f: *Ruminococcaceae*
OTU91	0.969	0.478	0.650	1.370	0.626	f: *Lachnospiraceae*
OTU17	0.035	0.041	2.446	0.006	1.441	f: *Ruminococcaceae*
OTU30	0.437	1.743	0.154	0.396	0.927	o: *Bacteroidales*
OTU21	3.219	0.012	0.071	0.089	0.106	g: *Streptococcus*
OTU22	2.097	0.089	0.083	0.673	0.461	f: *Lachnospiraceae*
OTU62	3.302	0.000	0.000	0.000	0.000	o: *Bacillales*
OTU24	1.311	0.750	0.230	0.744	0.089	f: *Prevotellaceae*
OTU85	0.012	1.270	0.809	0.071	0.727	g: *Acidaminococcus*
OTU42	2.694	0.000	0.000	0.000	0.000	o: *Lactobacillales*
OTU31	0.289	0.413	0.650	0.727	0.555	f: *Christensenellaceae*
OTU44	0.000	1.382	0.508	0.006	0.738	o: *Clostridiales*
OTU67	2.629	0.000	0.000	0.000	0.006	s: *Weissella salipiscis*
OTU28	0.030	0.691	0.496	0.951	0.431	f: *Lachnospiraceae*
OTU27	0.313	0.419	0.449	0.591	0.792	g: *Prevotella*

1 Thirty-seven OTUs with abundances higher than 0.5% in the microbial community were sorted from a total of 1409 OTUs, and defined as predominant OTUs.

2 The consensus sequence of each OTU was annotated to the closest lineage using the MOTHUR program against the SILVA 16S rRNA reference database. s: = species; g: = genus; f: = family; o: = order; c: = class; p: = phylum.

### Core Fecal Microbiota

The bacterial species in the feces of five rhinoceroses were further investigated for the presence of core gut microbiota (Table 3). The five libraries had 266 OTUs in common, which comprised 46.61%, 72.19%, 67.60%, 73.85%, and 68.16% of reads in X1, X2, X3, X4, and X5 libraries, respectively. Firmicutes and Bacteroidetes dominated in the shared OTUs, as well as the reads of shared OTUs. Of the total 1409 OTUs, the four libraries from healthy animals X2, X3, X4, and X5 had 350 OTUs in common, which comprised 85.60%, 78.40%, 83.55% and 82.34% of reads in each library, respectively (Table 4). The core microbiota were dominated by phyla Firmicutes and Bacteroidetes, represented by the *Ruminococcaceae*, *Lachnospiraceae*, *Rikenellaceae* and *Prevotellaceae* families (Table 5). In addition, 176 OTUs, which were only observed in the X1 library, were further analyzed. These X1-specific OTUs comprised 21.32% of total reads in the X1 library, and were dominated by Firmicutes and Actinobacteria phyla.

**Table 3:** Shared phyla among the 16S rRNA gene libraries from five rhinoceroses.

Phylum	Shared OTUs	Reads of shared OTUs					Reads of shared OTUs/Total reads (%)				
		X1	X2	X3	X4	X5	X1	X2	X3	X4	X5
Actinobacteria	4	50	36	30	29	31	0.30	0.21	0.18	0.17	0.18
Bacteroidetes	55	2,742	5,776	4,230	5,024	4,386	16.20	34.12	24.99	29.68	5.91
Chloroflexi	2	134	110	93	121	84	0.79	0.65	0.55	0.71	0.50
Firmicutes	189	4,745	5,986	6,899	6,918	6,884	28.03	5.36	40.75	40.86	0.66
Lentisphaerae	4	86	57	85	89	50	0.51	0.34	0.50	0.53	0.30
Planctomycetes	1	49	11	32	19	21	0.29	0.06	0.19	0.11	0.12
Proteobacteria	1	14	132	7	158	14	0.08	0.78	0.04	0.93	0.08
Spirochaetes	2	3	3	7	20	10	0.02	0.02	0.04	0.12	0.06
Tenericutes	1	8	50	11	8	15	0.05	0.30	0.06	0.05	0.09
Unclassified	7	60	60	50	116	43	0.35	0.35	0.30	0.69	0.25
Total shared sequences	266	7,891	12,221	11,444	12,502	11,538	46.61	72.19	67.60	73.85	68.16

**Table 4:** Shared phyla among the 16S rRNA gene libraries from four healthy rhinoceroses^1^.

Phylum	Shared OTUs	Reads of shared OTUs				Reads of shared OTUs/Total reads			
		X2	X3	X4	X5	X2	X3	X4	X5
Actinobacteria	7	59	44	48	57	0.35	0.26	0.28	0.34
**Bacteroidetes**	66	6,062	4,709	5,798	4,890	35.81	27.82	34.25	28.89
Candidate_division_TM7	1	10	3	2	5	0.06	0.02	0.01	0.03
Chloroflexi	2	110	93	121	84	0.65	0.55	0.71	0.50
**Firmicutes**	241	7,633	8,033	7,483	8,562	45.09	47.45	44.20	50.58
Lentisphaerae	10	294	206	249	128	1.74	1.22	1.47	0.76
Planctomycetes	1	11	32	19	21	0.06	0.19	0.11	0.12
Proteobacteria	4	142	20	176	18	0.84	0.12	1.04	0.11
Spirochaetes	4	16	10	31	14	0.09	0.06	0.18	0.08
Tenericutes	3	75	52	30	102	0.44	0.31	0.18	0.60
Unclassified	11	79	71	188	58	0.47	0.42	1.11	0.34
Total shared sequences	350	14,491	13,273	14,145	13,939	85.60	78.40	83.55	82.34

1 The phyla in bold letters represent core fecal microbiota.

**Table 5:** The predominant core microbiota (family level) in the samples from four healthy rhinoceroses^1^.

Family	Shared OTUs	Reads of shared OTUs				Reads of shared OTUs/Total reads			
		X2	X3	X4	X5	X2	X3	X4	X5
Lachnospiraceae	55	2,548	1,466	1,495	2,294	15.05	8.66	8.83	13.55
Prevotellaceae	7	1,626	1,128	1,348	1,391	9.06	6.66	7.96	8.22
Rikenellaceae	36	3,180	1,311	1,584	1,312	18.78	7.74	9.36	7.75
Ruminococcaceae	90	2,667	3,739	2,918	3,271	15.75	22.09	17.24	19.32
Total shared sequences	188	10,021	7,644	7,345	8,268	59.19	45.15	43.39	48.84

1 The core microbiota were generated from Table 4.

## Discussion

The microbial population in the hindgut plays a key role in the health and welfare of the herbivore [9]. An active and functional fibrolytic bacterial population in the hindgut converts fibrous feeds into volatile fatty acids which make a significant contribution to the energy requirements of the host [6]. So far, studies regarding the intestinal microbial flora of the white rhinoceros are relatively limited [10]. In the current study, the fecal bacterial community of the white rhinoceros has been determined comprehensively for the first time using high throughput sequencing technology. In the present study, it was not unexpected to find that a large number of bacteria in the feces of the white rhinoceros belonged to the unclassified genera based on the current database of 16S RNA gene sequences, since little work on this kind of wild herbivorous animal has been done before. However, to some extent, the result also reflects the weakness of high throughput sequencing that it is not precise for lower levels taxonomic classification because of the short-read lengths and background ‘noise' introduced by PCR and sequencing [11]. Nevertheless, the results might suggest that the white rhinoceros might possess specific intestinal microbiota for its special feeding habits. However, to fully understand these unknown bacteria and their special role to the hosts, further studies are still needed.

The white rhinoceros is an ungulate animal, with hoofs that have three toes on each foot. They are more closely related to horses (who are also ungulates) than hippos [12]. In addition, also like horses, rhinoceroses are hindgut fermenters with the ability to eat less nutritious vegetation than ruminants due to their faster digestion. In previous studies, the fecal bacterial communities of horses have been intensively investigated [4], [6], [13], [14]. Costa et al. compared the fecal microbiota of healthy horses and horses with colitis by high throughput sequencing, and found Firmicutes predominated among healthy horses, followed by Bacteroidetes and Proteobacteria [4]. Similar to the healthy horses, Firmicutes were also found to be the most predominant phylum in the feces of the five white rhinoceroses. This phylum has also been reported to be the most abundant in the hindgut of healthy humans and most of mammals [10], [15], [16]. Within the Firmicutes phylum, we found that the *Lachnospiraceae* and *Ruminococcaceae* families dominated in the feces of the five white rhinoceroses, which is consistent with previous studies on hindgut microbiota of humans and other mammals [17], [18]. Although most of these families were not classified at the genus and species levels, numerous bacteria such as *Ruminococcus* spp., *Butyrivibrio* spp., and *Clostridium* spp. are regarded as fiber-degraders in the rumen and the hindgut of herbivores [5], [6]. However, as another main fiber-degrader in the rumen, *Fibrobacter* was not detected in the feces of the white rhinoceros, likely due to our methodology. Considering that the bacterial communities in the hindgut of animals were believed to similar to those in feces [18], this result indicate that the fiber degrading bacteria in the hindgut of herbivores are dissimilar to those in the rumen. Nevertheless, the influence of the DNA extraction method used in this study could not be ignored.

Bacteroidetes is also one of the most abundant phyla in the gut of humans and herbivores [6], [15]. In the present study, this phylum was the second most abundant in the fecal bacterial community of all five white rhinoceroses, which is consistent with the finding in healthy horses and other mammals [4], [10]. However, in the rumen of dairy cows, Bacteroidetes was regarded as the most abundant phylum, which represented around 40–70% of abundance within the total bacterial community [5], [19]. The results indicate that unlike the ruminant, Bacteroidetes might play a lesser role in hindgut fermentation compared to the dominant Firmicutes phylum in non-ruminant herbivores. Nevertheless, we found that families *Rikenellaceae* and *Prevotellaceae* dominated in this phylum, which is also similar to the findings on other mammals [20], [21]. Interestingly, Bacteroidetes became predominant in the feces of horses affected by colitis. In contrast, the abundance of this phylum in the sample from X1 was much lower than in the samples from the four healthy animals. The possible reason for this variation may be the drug therapy for X1.

Noticeably, we found that the genus *Aerococcus* was predominant in the X1 library; in particular, *Aerococcus viridans*-related OTU was only found in the feces of rhinoceros X1 with the relative abundance of 4.62% within the total bacteria. *A. viridans* has been associated with different human infections, such as endocarditis, urinary tract infections, and meningitis [22]–[24]. In addition, this species has also been isolated from the milk of cows with subclinical mastitis [25], and from different clinical specimens of normally sterile body sites of pigs [26]. Moreover, *A. viridans* was found to be resistant to many antimicrobial drugs, including penicillins, cefotaxime, amikacin, trimethoprim/sulfamethoxazole, and glycopeptides [27]–[29], which is consistent with the fact that rhinoceros X1 had been treated with cefotaxime and amikacin due to constipation.

In this study, we found three female rhinoceroses had relatively high similar microbiota, although X3 was much older than X4 and X5, which suggests that the fecal microbiota of the rhinoceros might be influenced by the animal’s sex. This is consistent with the findings of a previous study where female and male macaques possessed distinctive microbiota in fecal and colonic contents [7]. Similarly, partitioning of the gut microbiota by sex has also been noted in mice [30]; however, the physiological mechanism such as circulating levels of hormones for the observed sexual dimorphism is unknown.

In the wider area of gut microbiology, there is active debate concerning the existence of a core stable microbiota. It is estimated that there are perhaps 5000 unique bacterial OTUs in the human gut when considered over a range of individuals under different spatial and temporal conditions [31]. However, it is speculated that there are perhaps 300 OTUs that make up the core stable microbial population in a healthy individual [32]. In the present study, 350 OTUs representing more than 75% of abundance within the total microbiota were regarded as core bacteria in four healthy rhinoceroses. Even when considering the outlier microbiota, the five rhinoceroses still had 266 core OTUs in the fecal microbiota. In addition, we found that the core bacteria in four healthy rhinoceroses were dominated by phyla Firmicutes and Bacteroidets including *Prevotellaceae*, *Rikenellaceae*, *Ruminococcaceae*, and *Lachnospiraceae* families, which is different from those reported by Ley et al for 106 mammals [10]. Costa et al found that only family *Lachnospiraceae* dominated the core bacterial population in the feces of healthy horses [4]. In the rumen of cows, the predominant core bacteria belonged to the *Prevotella* genus, *Lachnospiraceae* family, and the *Butyrivibrio* genus [5]. Possible reasons for the high percentage of core bacteria in the rhinoceros are that we evaluated only a few animals and the animals had the same diet in the same conditions. In addition, we removed the bacteria from fiber materials in the feces before DNA extraction, which can also affect the subsequent bacterial community using pyrosequencing analysis [33]. A higher diversity of predominant core bacteria in rhinoceroses compared with horses and cows might be responsible for its strong ability to adapt to the diet (e.g. toxic phytochemicals in the diet).

In summary, the work presented here describes the composition of the overall bacterial communities in the feces of five white rhinoceros living in a zoo. Our data reveals the presence of a complex bacterial community in the feces of the white rhinoceros. The rhinoceros possesses distinctive microbiota and core bacteria in the feces compared to horses. These observations increase our understanding of the bacterial ecosystem of this endangered animal, however, further study is still needed to know whether rhimoceroses in the wild have specific gut microbiota compared to other non-ruminant herbivores.

## Materials and Methods

### Collection of Fecal Samples

Five African white rhinoceroses were housed in the same room with a 3000-square meters outdoor playgroud at the Shanghai Wild Animal Park (see Table 6). X1 was treated with neostigmine bromide (0.6 g), cisapride (0.4 g), and rhubarb-soda tablet (50 g) through oral administration accompanying with intramuscular injection of cefotaxime (20 g) and amikacin (4 g) twice per day for six days because of the bad appetite and constipation, and had recovered one month prior to the study. X2, X3, X4, and X5 were healthy animals. The twice-daily diet for each animal consisted of 100–125 kg fresh, local grass (mainly gramineous pasture including *Digitaria* spp., *Eleusine indica* and *Setaria viridis*), 7.5 kg hay pellets (*Leymus chinensis*), and 2 kg carrot. In September 2011, fresh fecal sample (approximate 100 g) were immediately collected by the animal raiser when each animal was upon defecation in the morning, sent to the laboratory in foamed plastic containers with dry ice, and processed immediately after arrival. The samples were pretreated according to Wang et al. [34] and Wei et al. [35] as follows: 50 g of feces was suspended in a sterile plastic beaker containing 250 ml of sterile phosphate-buffered saline (PBS) (0.05 mol/l, pH 7.4). The sample was stirred with a sterile plastic rod for about 30 min to remove the bacteria from the plant residue. The suspension then was divided into 60-ml aliquots and transferred to eight sterile 80-ml centrifuge tubes and vortexed vigorously for 15 min. The samples were centrifuged at 200 g for 5 min three times (each time the supernatant was transferred to a new tube) to remove coarse particles. The cells in the supernatant were collected and washed three times by centrifuging at 9000 g for 3 min with 30 ml fresh PBS. Finally, the washed cell pellets were re-suspended in one tube in 10 ml of sterile PBS, divided into 1-ml aliquots, and stored at −20°C for DNA extractions within one week.

**Table 6:** Information on the rhinoceroses used in this study.

Rhinoceros	Sex	Age (years)	Health condition
X1	male	26	treated with neostigmine bromide, cisapride and rhubarb-soda tablet through oral administration accompanying with intramuscular injection of antibiotic therapy one month prior to the study
X2	male	26	healthy
X3	female	25	healthy
X4	female	17	healthy
X5	female	17	healthy

### Ethics Statement

The study was approved by Nanjing Agricultural University Animal Care and Use Committee. Fecal samples of the rhinoceros were collected with the permission of Chunzhong Xu, the director of Shanghai Wild Animal Park. The study did not involve endangered or protected species.

### DNA Extraction

The total genomic DNA was isolated from the pretreated fecal samples using the commercially available stool DNA extraction kit according to the instructions of the manufacturer (QIAamp DNA Stool Mini Kit, Qiagen, Hilden, Germany). The concentration of extracted DNA was determined using a Nano-Drop 1000 spectrophotometer (Thermo Scientific Inc., Wilmington, DE, USA).

### PCR Amplification, Amplicon Quantitation, and Pyrosequencing

To analyze the taxonomic composition of the bacterial community, universal primers (8F 5′-AGA GTT TGA TCC TGG CTC AG-3′ and 533R 5′-TTA CCG CGG CTG CTG GCA C-3′) targeting the V1–V3 region of 16S rRNA gene were chosen for the amplification and subsequent pyrosequencing of the PCR products [36]. The PCR were carried out in triplicate with: 50 ml reactions with 10 µl 5-fold reaction buffer, 50 ng of DNA, 0.4 mM each primer, 0.5 U Pfu polymerase (TransStart-FastPfu DNA Polymerase, TransGen Biotech), and 2.5 mM dNTPs. The amplification program consisted of an initial denaturation step at 94°C for 4 min. This was followed by 25 cycles, where 1 cycle consisted of 94°C for 30 s (denaturation), 55°C for 30 s (annealing), 72°C for 30 s (extension), and a final extension of 72°C for 10 min. All PCR products were visualized on agarose gels (2% in TBE buffer) containing ethidium bromide, and purified with a DNA gel extraction kit (Axygen, China).

Prior to sequencing, the DNA concentration of each PCR product was determined using a Quant-iT PicoGreen double-stranded DNA assay (Invitrogen, Germany), and was quality controlled on an Agilent 2100 bioanalyzer (Agilent, USA). Amplicon pyrosequencing was performed from the A-end using a 454/Roche A sequencing primer kit on a Roche Genome Sequencer GS-FLX Titanium platform at Majorbio Bio-Pharm Technology Co., Ltd., Shanghai, China.

### Bioinformatics Analysis

The end fragments were blunted and tagged on both ends with ligation adaptors that contained a unique 10-bp sequence (sample specific barcode sequence) and a short 4-nucleotide sequence (TCAG) called sequencing key, which were recognized by the system software and the priming sequences. All pyrosequencing reads were filtered according to barcode and primer sequences. The resulting sequences were further screened and filtered for quality. Sequences that were shorter than 200 bp in length, contained ambiguous characters, contained over two mismatches to the primers, or contained mononucleotide repeats of over six nt were removed. To assess bacterial diversity among samples in a comparable manner, a randomly selected, 16929-sequence (the lowest number of sequences in the five samples) subset from each sample was aligned using the ‘align.seqs’ command and compared with the Bacterial SILVA database (SILVA version 108; http://www.arb-silva.de/documentation/background/release-108/). The aligned sequences were clustered into operational taxonomic units (OTUs) defined by 97% similarity [37] using CD-HIT-OUT program [38]. We also calculated the coverage percentage using Good’s method [39], abundance based coverage estimator (ACE), bias-corrected Chao richness estimator, and the Shannon and Simpson diversity indices using the MOTHUR program (http://www.mothur.org) [40]. Libshuff analysis was used to compare population structure between different aminals. The heatmap figure was generated using custom Perl scripts. The raw pyrosequencing reads were submitted to Sequencing Read Archive (SRA) database under the accession id: SRA073469.
